# Epidemiology and antimicrobial resistance profile of *Neisseria gonorrhoeae* in Catalonia, Spain, 2016–2019

**DOI:** 10.1007/s10096-023-04601-0

**Published:** 2023-05-10

**Authors:** Mercè Herrero, Sonia Broner, Adrià Cruells, Silvia Esteve, Lourdes Ferré, Jacobo Mendioroz, Mireia Jané, Pilar Ciruela, the working group at MRSC, Miguel Ángel Benítez, Jordi Bosch, Cristina Pitart, Frederic Ballester, Ana Calderón, Teresa Falgueras, Carmina Martí, Mª Àngeles Pulido, Margarida Curriu, Ester Sanfeliu, Percy Juan Ayala, Carme Gallés, Elisenda Capdevila, Pilar Hernández, Paula Gassiot, Carme Mora, Frederic Gómez, Araceli González, Màrius Juanpere Aixalà, Eduardo Padilla, Amadeu Gené, Ferran Navarro, Alba Rivera, Ferran Sánchez, Gloria Trujillo, Joan López, Montserrat Olsina, Pepa Pérez, Mar Olga Pérez, Joan Manel Ramírez, Xavier Raga, Judith Lucena, Jesús Aramburu, Esther Sanfeliu Riera, Goretti Sauca, Inés Valle, Anna Vilamala, Yannick Hoyos, Jordi Cámara, Jordi Niubó, Graciela Rodríguez, Fe Tubau, Maria Dolores Quesada, Nuria Torrellas, Natàlia Claver, Teresa Bastida, Rosalia Santos, Olga González-Moreno

**Affiliations:** 1 Subdirectorate General for Public Health Surveillance and Emergency Response, Catalan Public Health Agency, Government of Catalonia, 08005 Barcelona, Spain; 2 Research Support Unit of Central Catalonia, Jordi Gol i Gurina University Research Institute for Primary Health Care, 08272 Sant Fruitós de Bages, Spain; 3 CIBER Epidemiologia y Salud Pública (CIBERESP), Carlos III Health Institute, 28029 Madrid, Spain; 4 Department of Medicine, University of Barcelona, Barcelona, Spain

**Keywords:** *Neisseria gonorrhoeae*, Multi-drug resistant, Ceftriaxone, Azithromycin, Antimicrobial resistance

## Abstract

Antimicrobial resistance data for *Neisseria gonorrhoeae *is globally sparse and resistant strains are emerging in Catalonia. We aim to describe epidemiological and antimicrobial resistance in all patients infected with *N. gonorrhoeae* during the period from 2016 to 2019, using available antimicrobial susceptibility data. We retrospectively analysed confirmed *N. gonorrhoeae* cases notified to Catalonia’s microbiological reporting system. Antibiotic susceptibility testing (azithromycin, cefixime, ceftriaxone, ciprofloxacin, penicillin, spectinomycin, and tetracycline) was assessed using clinical breakpoints published by the European Committee on Antimicrobial Susceptibility Testing. Incidence rates were calculated and proportions were compared using the χ^2^ test or Fisher’s exact test, and analysed using the Statistical Package for Social Sciences (SPSS 18.0). A total of 14,251 confirmed cases of *N. gonorrhoeae* were notified. Incidence increased from 30.7 cases/100,000 person-years (*p* < 0.001) in 2016 to 64.7 in 2019. Culture was available in 6,292 isolates (44.2%), of which 5,377 (85.5%) were resistant to at least one of the antibiotics tested. Azithromycin resistance rose from 6.1% in 2016 to 16% in 2019 (*p* < 0.001). Only 1.0% (45 cases) were resistant to ceftriaxone. Multidrug-resistant *N. gonorrhoeae* increased from 0.25% in 2016 to 0.42% in 2019 (*p* = 0.521). One case presented extensively drug-resistant *N. gonorrhoeae*. In Catalonia, 10% of the *N. gonorrhoeae* isolates were resistant to azithromycin in the 2016–2019 period. According to World Health Organization guidelines, resistance above 5% indicates an alert to review treatment guidelines. Antimicrobial susceptibility testing in clinical practice followed by surveillance and interventions are essential to monitor trends and prevent the spread of antimicrobial resistance.

## Introduction

Gonorrhoea is one of the most common sexually transmitted bacterial infections (STI) and an important cause of morbidity worldwide. Without treatment, the disease can cause severe complications such as pelvic inflammatory disease and ectopic pregnancy in women, and infertility in both genders. In addition, neonatal gonococcal conjunctivitis may lead to scarring and blindness [1].

Laboratory diagnosis is determined by gram staining, conventional agar-based culture method, and/or nucleic acid amplification test (NAAT). In the case of gonococcus, it is especially important to obtain a sample of urethral/cervical exudate for culture before starting treatment; this sample will be used to perform an antibiogram, confirm the appropriate treatment and track changes in antimicrobial resistance (AMR) [2].

Antimicrobial resistance is a major public health problem. Systematic epidemiological surveillance of AMR to *N. gonorrhoeae* is essential for several reasons. First, to detect emerging resistance to antimicrobials in strains circulating among the population; second, to contribute to the design of action plans for the prevention and control of gonorrhoea infection, and third, to allow professionals to evaluate its extension worldwide and report on local and global strategies to control AMR to gonococcal disease. Current surveillance is often suboptimal and presents many challenges, especially in countries with the highest burden of resistance [3].

Treatment of gonococcal infection is currently affected by the appearance of strains resistant to almost all groups of antibiotics used, as well as by the recent reduced susceptibility appearing worldwide in third-generation cephalosporins [4].

In order to reduce the risk of resistance to the use of antibiotic monotherapy, the World Health Organization (WHO) recommends that local resistance data determine the choice of therapy (both dual therapy and monotherapy). In territories where local resistance data are unavailable, the guidelines suggest combining azithromycin and ceftriaxone-based therapy or, alternatively, treating with cefixime prior to monotherapy [5].

Since 2009, the European Centre for Disease Prevention and Control (ECDC) has been coordinating the Gonococcal Antimicrobial Surveillance Programme (Euro-GASP), a specific epidemiological surveillance programme for antibiotic resistance in *Neisseria gonorrhoeae*. Funded by an international network, the programme is led by Public Health England in the UK and includes the Örebro University Hospital in Sweden [6].

According to the ECDC, incidence rates (IR) of gonococcal infection have increased significantly in Europe over the years, rising from 8.7 cases/100,000 persons in 2010 to 18.9/100,000 in 2016; 46% of these cases were registered as men who have sex with men [6]. In Spain, the gonococcal infection incidence has increased from 2.02 cases/100,000 persons in 2001 to 13.89/100,000 in 2016. Of 6,456 notified cases, 83.7% occurred in males, indicating a male/female ratio of 5:1.

With respect to age groups, the highest incidence occurs in 20 to 24-years-old (89.2/100,000 in males and 21.5/100,000 in women) in Europe and worldwide. In 2016, the highest IR in Spain was in Catalonia, followed by Asturias and Madrid [7]. In Barcelona, between 2013 and 2017, the observed proportions of cases resistant to first-line treatments were 4.9% for cefixime, 0.9% for ceftriaxone, and 3.0% for azithromycin. However, the proportion of isolates showing resistance to all antibiotics was approximately 1% of cases [8].

In Catalonia, the Epidemiological Surveillance Network (Decree 203/2015 [9], of 15 September) was created in 2015 to regulate reporting systems for notifiable diseases and epidemic outbreaks, and establish a microbiological reporting system for Catalonia (MRSC) as a notifiable system. Through the MRSC, microbiologists notify confirmed cases of acute illnesses, enabling the study of microorganisms with antibiotic susceptibility and notifiable resistance profiles.

In this study, we aim to describe the epidemiological characteristics of confirmed cases of *N. gonorrhoeae* and antimicrobial susceptibility notified to the MRSC between 2016 and 2019.

## Methods

### Study population

The study population considered all patients notified to the MRSC who received treatment in hospitals and primary health care centres in Catalonia or presented with a new confirmed episode of acute infection by *N. gonorrhoeae* between 2016 and 2019.

### Data collection

All data was processed by the Subdirectorate General for Public Health Surveillance and Emergency Response at the Catalonian Public Health Agency, including the introduction, validation, cleaning and analysis of data records according to the diagnostic criteria, ensuring that cases met the confirmed case definition in line with established criteria. The epidemiological, laboratory and clinical data collected included the age and sex of the patient, anatomical site of infection, previous gonorrhoea diagnosis, diagnostic technique, type of centre, coinfection with other STIs, and antibiotic sensitivity in isolated strains. The site of infection was classified as urethral/balanopreputial, vaginal/endocervix, pharynx, anorectal, urine and others (broadly categorized under genital/anorectal area and non-genital area). The age groups were split as follows: 0–11 years, 12–19 years, 20–29 years, 30–39 years, 40–49 years, and ≥ 50 years.

A case was considered laboratory-confirmed when at least one of the following diagnostic criteria was met [10]:

- isolation of *N. gonorrhoeae* in a clinical sample
- detection of nucleic acids from *N. gonorrhoeae* in a clinical sample
- observation of intracellular gram-negative diplococcus obtained in a smear of male’s urethral material (direct microscopy)

### Data analysis

A descriptive analysis of the study variables was conducted. With regard to statistical analysis, IR per 100,000 person-years were calculated according to demographic data delivered by the Institute of Statistics of Catalonia [11] for the studied age groups and sex. Proportions were compared using the χ^2^ test or Fisher’s exact test, as appropriate. Incidence rates were calculated and changes were assessed according to IR ratios and expressed as the percentage change in incidence. Two-sided *p*-values < 0.05 were considered statistically significant. Analyses were conducted using the Statistical Package for Social Sciences (SPSS 18.0).

### Antimicrobial susceptibility

The determination of antimicrobial susceptibility testing was studied for seven antibiotics: azithromycin, cefixime, ceftriaxone, ciprofloxacin, penicillin, spectinomycin, and tetracycline. The laboratories used an “antibiogram kit”; where not available, manual susceptibility testing was performed for each antibiotic. For the study of resistance for each antibiotic, the breakpoints recommended by the European Committee on Antimicrobial Susceptibility Testing (version 6.0) were used, with the consensus of the MRSC working group included in the Protocol for the Surveillance of Antimicrobial Resistance in Catalonia [12]. The clinical breakpoints (susceptible, resistant) were as follows: azithromycin (MIC ≤ 0.25 mg/L, MIC > 0.5 mg/L); ceftriaxone and cefixime (MIC ≤ 0.125 mg/L, MIC > 0.125 mg/L); ciprofloxacin (MIC ≤ 0.03 mg/L, MIC > 0.06 mg/L); penicillin (MIC ≤ 0.06 mg/L, MIC > 1.0 mg/L); spectinomycin (MIC ≤ 64 mg/L, MIC > 64 mg/L); and tetracycline (MIC ≤ 0.5 mg/L, MIC > 1.0 mg/L). The production strains of beta-lactamase were also analysed.

The criteria for this study were adapted from Tapsall et al. (2009), when definitions were established for multidrug-resistant gonococci (MDR-NG) and extensively drug-resistant gonococci (XDR-NG) [13].

Multi-drug resistant gonococci are strains resistant to one category I antibiotic – extended spectrum cephalosporins (ESC) or spectinomycin – plus at least two other antibiotics in category II (penicillin, ciprofloxacin and azithromycin). Extensively drug-resistant gonococci strains are resistant to two or more category I antibiotics (ESC and spectinomycin); and to three or more in category II (penicillin, ciprofloxacin, azithromycin, aminoglycosides and carbapenem).

In our definition of MDR-NG, we used cefixime and ceftriaxone as ESC or XDR-NG and did not consider aminoglycosides and carbapenem.

We reviewed patient files for studied cases presenting multi-drug resistant and extensively drug-resistant gonococci.

**Co-infections** with other STIs were studied, considering a three-week window period between the gonorrhoea infection and other STIs. Analyses of all cases of *N. gonorrhoeae* (14,251) were conducted to determine whether other STIs (chlamydia, trichomonas, herpes or syphilis) were present during the three-week period from the initial diagnosis of *N. gonorrhoeae.*

**Reinfection** episodes were studied for each confirmed case of *N. gonorrhoeae* subsequent to the first infection. *N. gonorrhoeae* positive tests within 60 days of a previous positive confirmed case were considered a single episode.

## Results

Between 2016 and 2019, a total of 14,251 confirmed cases of *N. gonorrhoeae* were reported in Catalonia. In Table 1, we summarize the case incidence according to year, age group and sex; and diagnosis technique, anatomical site of infection and origin of the sample (clinical setting).

**Table 1:** Characteristics of confirmed cases of *Neisseria gonorrhoeae*. Catalonia, Spain, 2016−2019 (*n* = 14,251 cases)

Characteristics	2016 (*n*=2,308)			2017 (*n*=3,319)			2018 (*n*=3,659)			2019 (*n*=4,965)			Total* (*n*=14,251)		
	*n*	%	IR	*n*	%	IR	*n*	%	IR	*n*	%	IR	*n*	%	IR
Age Group*															
0−11 years	4	0.2	0.4	1	0.0	0.1	1	0.0	0.1	7	0.1	0.8	13	0.1	0.3
12−19 years	160	6.9	27.6	212	6.4	35.6	297	8.1	48.4	391	7.9	61.5	1,060	7.4	43.7
20−29 years	948	41.1	121.9	1,466	44.2	187.7	1,487	40.6	187.9	1,964	39.6	242.5	5,865	41.2	185.6
30−39 years	743	32.2	63.9	1,023	30.8	91.4	1,088	29.7	100.7	1,488	30.0	141.0	4,342	30.5	98.3
40−49 years	316	13.7	25.3	443	13.3	35.0	549	15.0	42.8	777	15.6	59.8	2085	14.6	40.9
≥ 50 years	137	5.9	4.9	173	5.2	6.1	235	6.4	8.1	335	6.7	11.4	880	6.2	7.7
Total	2,308	100	30.7	3,319	100	43.9	3,659	100	48.1	4,965	100	64.7	14,251	100	46.9
Sex															
Women	402	17.4	10.5	597	18	15.5	742	20.3	19.2	1,116	22.5	28.6	2,857	20	18.5
Men	1,906	82.6	51.6	2,722	82	73.4	2,917	79.7	78.2	3,849	77.5	102.1	11,394	80	76.4
Diagnosis															
Culture	1,101	47.7		1,346	40.6		1,713	46.8		2,132	42.9		6,292	44.2	
PCR	1,207	52.3		1,970	59.4		1,946	53.2		2,832	57		7,955	55.8	
Direct microscopy	0	0.0		3	0.0		0	0.0		1	0.0		4	0.0	
Specimen**															
Urethral/balanopreputial	1,254	47.0		1,724	44.9		1,942	47.4		2,261	40.4		7,181	44.3	
Anorectal	418	15.7		626	16.3		626	15.3		900	16.1		2,570	15.9	
Endocervical/vaginal	369	13.8		522	13.6		664	16.2		941	16.8		2,496	15.4	
Pharynx	377	14.1		628	16.4		454	11.1		909	16.2		2,368	14.6	
Urine	222	8.3		317	8.3		374	9.1		538	9.6		1,451	9.0	
Others***	29	1.1		22	0.6		35	0.9		50	0.9		136	0.8	
Clinical setting															
Hospital	877	38		1,368	41.2		2,173	59.4		2,951	59.4		7,369	51.7	
Primary care	1,431	62		1,951	58.8		1,486	40.6		2,014	40.6		6,882	48.3	

*IR* incidence rate per 100,000 habitants

*In 6 cases age was unknown

**Some cases have more than one specimen

***Genital - anorectal area [Abscess (1); Penis abscess (1); Bartholin abscess (2); Endometrium (3); Douglas sack exudate (4); Genital swab (4); Glans (2); Penis (1); Pelvis pus (1); Genital ulcer (3); Genital chancre (27); Sperm (26)]. Non-genitalia area [Lymph node biopsy (1); Conjunctival exudate (7); Non-surgical exudate (2); Articular fluid (4); Peritoneal fluid (3); Blood (1); Conjunctival secretion (4); Serum (38)]; Not informed (1)

Overall incidence was 46.9 cases/100,000 persons/year, with an increase of 110.7% in 2019 compared to the rate in 2016 (IR: 30.7 cases/100,000 persons/year in 2016; and 64.7 cases/100,000 persons/year in 2019 (*p* < 0.001).

Age and sex were available for almost all cases (age was missing in only six cases). Over 80% of cases were males. Overall ratio of male cases to female cases was 4:1, with this distribution exhibiting a diminishing trend over time, from 4.7:1 in 2016 to 3.4:1 in 2019 (*p* < 0.001). The incidence rose by 98% in males and by 172% in females between 2016 and 2019. The median age was 30 years (with a range of 0 to 89 years), with 31 years for males and 25 years for females. Sex and age distributions were similar throughout the period, with a high increase in female incidence in 2019 compared to 2016 (10.5 cases per 100,000 persons in 2016; and 28.6 cases/100,000 persons in 2019; *p* < 0.001). The highest incidence occurred in the 20–29 age group (185.6 cases/100,000 persons/year), followed by the 30–39 age group (98.3 cases/100,000 persons/year).

**Fig. 1:**
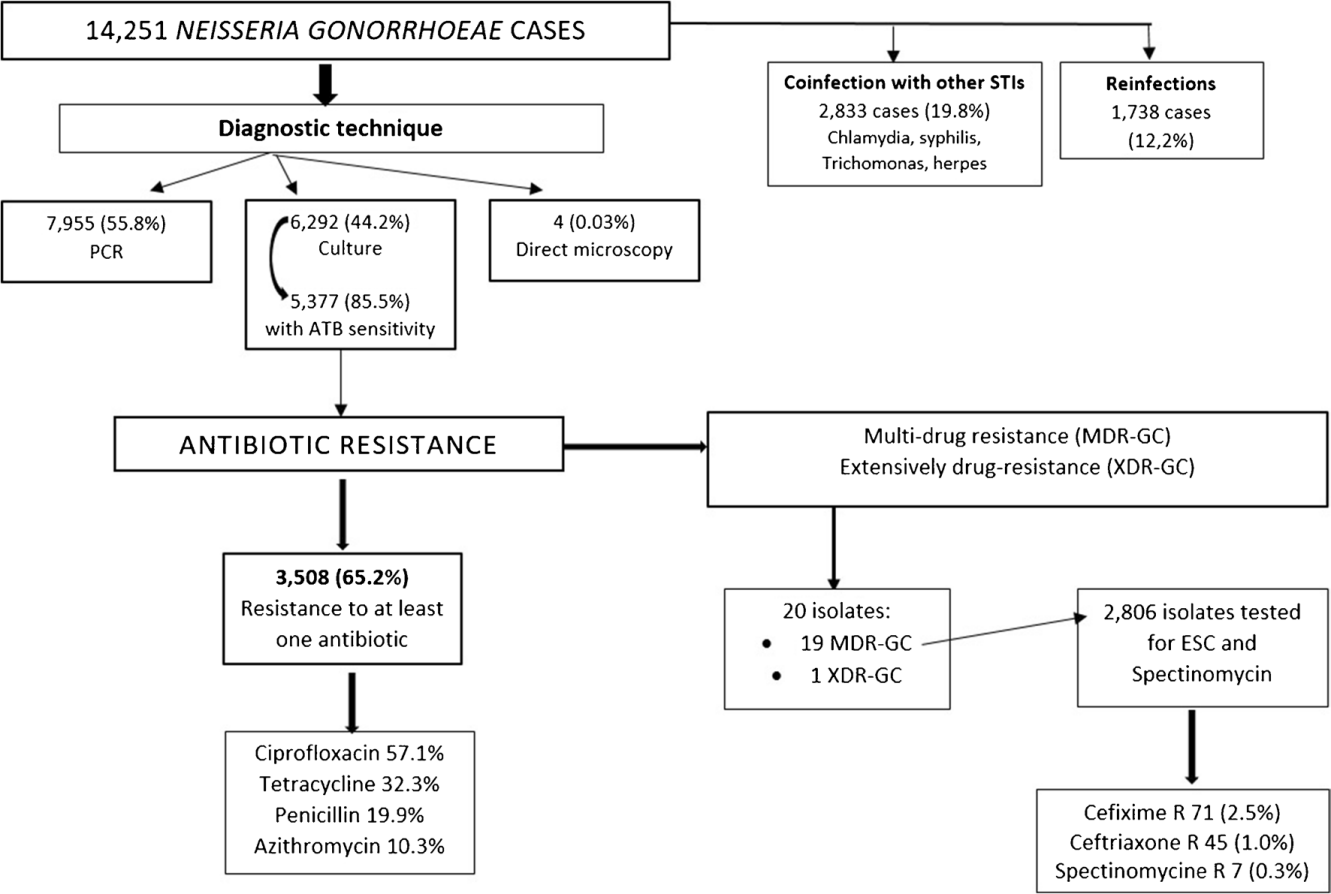
Flowchart for determining index events for inclusion in outcome analyses. Catalonia, Spain, 2016–2019. Abbreviations: ATB, Antibiotic; PCR, Polymerase chain reaction; ESC, extended-spectrum cephalosporins; R, resistant

Regarding diagnostic technique, culture was available in 6,292 cases (44.2%); 7,955 (55.8%) cases were analysed only by PCR and 4 of these (0.03%) were analysed by direct microscopy (Table 1 and Fig. 1). Laboratory culture decreased slightly, from 47.7% in 2016 to 42.9% in 2019, but not significantly, due to the shift from use of bacterial culture to NAAT for diagnosis of gonorrhoea*.* Of 16,202 samples, 12,322 (76.1%) were of genital-anorectal origin, 2,368 samples (14.6%) were pharyngeal, 1,451 samples (9.0%) were from urine, and 61 samples (0.4%) were from other anatomical site. In one case, site of infection was not reported. Confirmed cases of gonococcal infection came both from primary care (48.3%) and hospital care (51.7%).

### Antimicrobial susceptibility and resistance profiles of *Neisseria gonorrhoeae* isolates

The antimicrobial susceptibility study was carried out on 5,377 (85.5%) strains of 6,292 culture-diagnosed cases (Fig. 1). The percentage of antimicrobial susceptibility studied among culture-diagnosed cases and declared to the public system has increased in recent years, from 74.5% in 2016 to 90.1% in 2019 (*p* < 0.001).

The antibiotic with the highest number of isolates studied was ciprofloxacin (5,315/6,292 or 84.5%); followed by penicillin (5,165/6,292 or 82.1%); azithromycin (4,401/6,292 or 69.9%); ceftriaxone (4,361/6,292 or 69.3%); cefixime (2,806/6,292 or 44.6%); spectinomycin (2,290/6,292 or 36.4%) and tetracycline (2,028 /6,292 or 32.2%). Almost 50% of the isolates were tested for five antibiotics and close to 26% for six or seven antibiotics.

Of 5,377 *N. gonorrhoeae* isolates tested for antibiotic sensitivity, 3,508 (65.2%) presented resistance to at least one of the antibiotics analysed. The prevalence of resistance among the isolates tested for ciprofloxacin was 57.1% (3,033/5,315). Among those tested for tetracycline, penicillin and azithromycin, prevalence was 32.3% (656/2,028), 19.9% (1,027/5,165), and 10.3% (455/4,401), respectively. Between 2016 and 2019, percentages of resistance to ciprofloxacin, tetracycline and penicillin were relatively high and stable. Of the 4,361 strains analysed against ceftriaxone, 45 (1.0%) were resistant, while 71 (2.5%) out of 2,806 strains were resistant to cefixime. Seven (0.3%) out of 2,290 strains were resistant to spectinomycin (Table 2).

**Table 2:** Antimicrobial susceptibility in *Neisseria gonorrhoeae* isolates. Catalonia, Spain, 2016 − 2019

Antimicrobials	Percentage of*: Susceptible/Susceptible, increase exposure/Resistant				
	2016	2017	2018	2019	Total
Ceftriaxone	99.5/NA/0.5	98.5/NA/1.5	98.9/NA/1.1	99.1/NA/0.9	99.0/NA/1.0
	(*n* = 645)	(*n* = 888)	(*n* = 1,220)	(*n* = 1,608)	(*n* = 4,361)
Cefixime	97.0/NA/3	95.4/NA/4.6	97.7/NA/2.3	98.1/NA/1.9	97.5/NA/2.5
	(*n* = 164)	(*n* = 434)	(*n* = 1,042)	(*n* = 1,166)	(*n* = 2,806)
Spectinomycin	99.8/NA/0.2	100/NA/0	99.3/NA/0.7	99.3/NA/0.7	99.7/NA/0.3
	(*n* = 576)	(*n* = 821)	(*n* = 460)	(*n* = 433)	(*n* = 2,290)
Ciprofloxacin	44.7/0.7/54.5	45.9/2.3/51.8	42.5/1.0/56.5	37.1/1.2/61.7	41.6/1.3/57.1
	(*n* = 807)	(*n* = 1.120)	(*n* = 1,492)	(*n* = 1,896)	(*n* = 5,315)
Penicillin	10.5/67.6/21.9	10/74.4/15.6	11.1/68.3/20.6	13.2/65.9/21.0	11.5/68.6/19.9
	(*n* = 802)	(*n* = 1,080)	(*n* = 1,429)	(*n* = 1,854)	(*n* = 5,165)
Tetracycline	38.5/28.2/33.3	45.7/23.4/30.9	54.8/12.8/32.4	49.6/17.7/32.7	48.7/19.0/32.3
	(*n* = 291)	(*n* = 411)	(*n* = 564)	(*n* = 762)	(*n* = 2,028)
Azithromycin	89.2/4.8/6.1	89.4/4.9/5.8	81.3/10.4/8.3	77.6/6.4/16.0	82.7/7.0/10.3
	(*n* = 627)	(*n* = 903)	(*n* = 1,217)	(*n* = 1,654)	(*n* = 4,401)

Abbreviations: NA, not applicable

^*^The clinical breakpoints (susceptible, resistant) were as follows: ceftriaxone and cefixime (MIC ≤ 0.125 mg/L, MIC > 0.125 mg/L); ciprofloxacin (MIC ≤ 0.03 mg/L, MIC > 0.06 mg/L); spectinomycin (MIC ≤ 64 mg/L, MIC > 64 mg/L); penicillin (MIC ≤ 0.06 mg/L, MIC > 1.0 mg/L); tetracycline (MIC ≤ 0.5 mg/L, MIC > 1.0 mg/L); azithromycin (MIC ≤ 0.25 mg/L, MIC > 0.5 mg/L)

Of the strains with penicillin sensitivity data (*n* = 5,165), beta-lactamase production data was available in 2,187 (42.3%). Of the penicillin-resistant strains (*n* = 1,027), only 410 (39.9%) yielded beta-lactamase production data and, of these, 298 (72.7%) were positive for beta-lactamase.

**Fig. 2:**
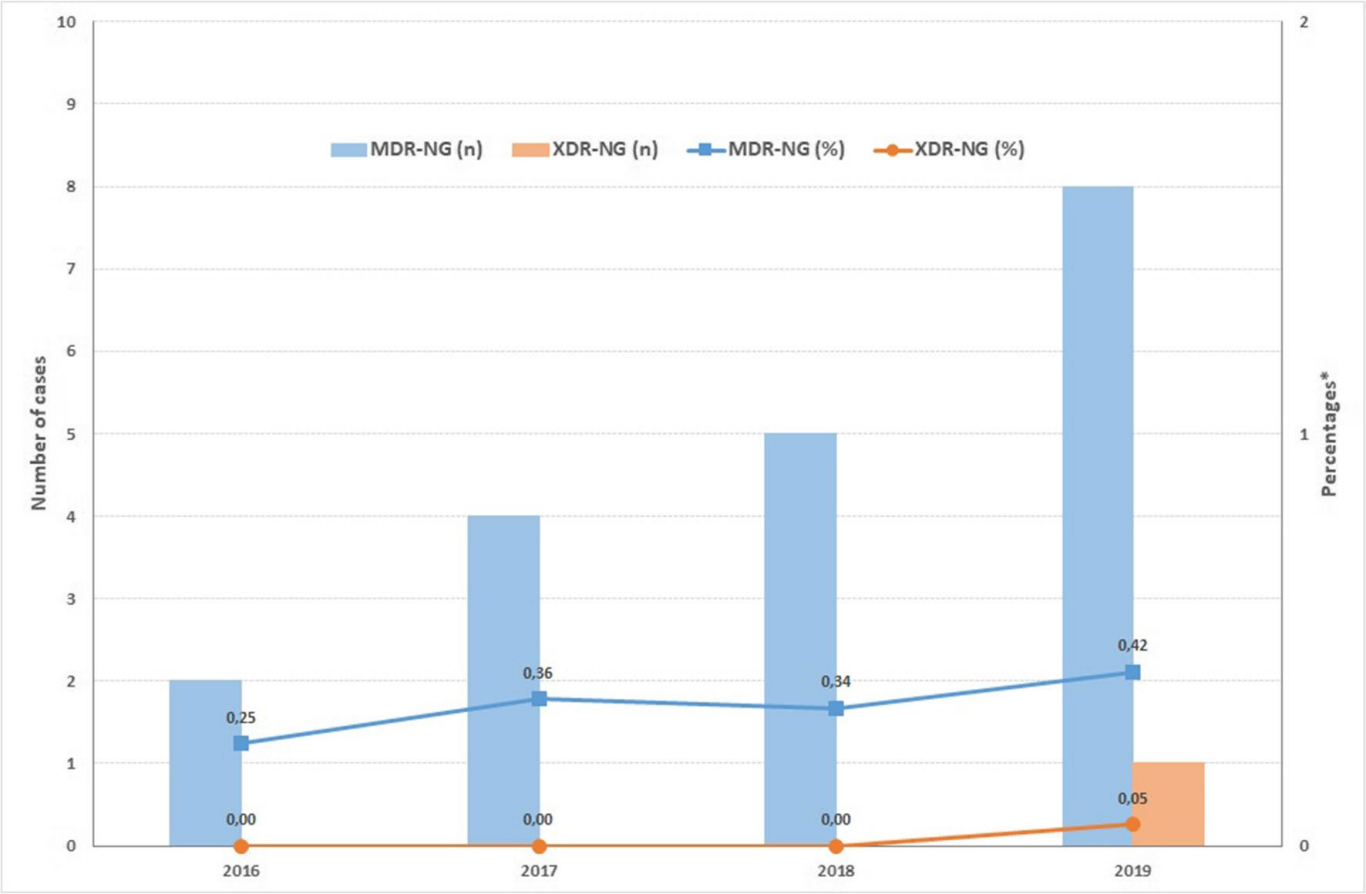
Multidrug-resistant and extensively drug-resistant *Neisseria gonorrhoeae*. Catalonia, Spain, 2016–2019. Abbreviations: n, number; MDR-NG, multidrug-resistant gonococci; XDR-NG, extensively drug-resistant gonococci. *Percentages based on total number of isolates of ceftriaxone by year (2016: 807 cases; 2017: 1,120 cases; 2018: 1,492 cases; 2019: 1,896 cases)

Resistance to azithromycin rose significantly over time, from 6.1% in 2016 to 16% in 2019 (*p* < 0.001). However, resistance to ceftriaxone did not increase significantly, from 0.5% in 2016 to 0.9% in 2019 ( *p* = 0.793). For cefixime, overall resistance decreased from 3% in 2016 to 1.9% in 2019 ( *p* = 0.013), as shown in Table 2, Fig. 2.

#### Multi-resistant strains

A total of 19 cases (0.4%) were identified as multi-drug resistant *N. gonorrhoeae* (MDR-NG); 79% of these occurred in males and 21% in females (Figs. 1 and 2).

#### Extensively drug-resistant

***N. gonorrhoeae*** (XDR-NG). Only one case presented XDR-GC in 2019. The case was resistant to ceftriaxone, cefixime, azithromycin, penicillin and tetracycline and was only sensitive to spectinomycin (Figs. 1 and 2).

When the characteristics of the 20 patients with MDR-NG and XDR-NG were further analysed, twelve of the patients were of Spanish origin, three were from Morocco, two from South America and three patients had no recorded origin. The median age of these patients was 31 years old (with a range from 22 to 60 years) (Figs. 1 and 2).

#### Co-infection of gonorrhoea with other STIs

We found other STIs present in 2,833 (19.9%) of 14,251 cases of gonococcal infection (Fig. 1). Co-infection increased slightly between 2016 and 2019 (from 19.7% to 21%). *Chlamydia trachomatis* was the most frequent co-infection (86.0%), followed by *Treponema pallidum* (8.5%), *Trichomonas vaginalis* (3.6%), and the herpes virus (1.9%). Females were more likely to be co-infected than males (30.4% versus 15.1%; *p* < 0.001) for all microorganisms, but not for *Treponema pallidum* (0.3% versus 2.0%; *p* < 0.001).

#### Reinfection

Reinfection rates were 12.2% (1,738 out of 14,251 cases) for the study period from 2016 to 2019. Among males, the median time since the first infection was 12.2 months (0.3–46 months), compared to 7.4 months (1.7–36 months) among females and globally, reinfections ocurred more in males than in females (7.2 versus 1.8%) (Table 3).

**Table 3:** Number of *Neisseria gonorrhoeae* reinfections in one-year period since first episode by sex. Catalonia, Spain, 2016–2019

	2016		2017		2018		2019		Total	
	F	M	F	M	F	M	F	M	F	M
	*n* (%)	*n* (%)	*n* (%)	*n* (%)	*n* (%)	*n* (%)	*n* (%)	*n* (%)	*n* (%)	*n* (%)
All cases	402	1,906	597	2,722	742	2,917	1,116	3,849	2,857	11,394
Time to next infection										
< 3 mo	2 (0.5)	27 (1.4)	3 (0.5)	29 (1.1)	2 (0.3)	16 (0.5)	6 (0.5)	43 (1.1)	13 (0.5)	115 (1.0)
3-< 6 mo	2 (0.5)	26 (1.4)	2 (0.3)	87 (3.2)	5 (0.7)	58 (2.0)	11 (1)	93 (2.4)	20 (0.7)	264 (2.3)
6-< 9 mo	0 (0.0)	27 (1.4)	2 (0.3)	83 (3)	2 (0.3)	43 (1.5)	10 (0.9)	84 (2.2)	14 (0.5)	237 (2.1)
9-< 12 mo	0 (0.0)	6 (0.3)	1 (0.2)	57 (2.1)	1 (0.1)	58 (2)	2 (0.2)	81 (2.1)	4 (0.1)	202 (1.8)
Total	4 (1.0)	86 (4.5)	8 (1.3)	256 (9.4)	10 (1.3)	175 (6)	29 (2.6)	301 (7.8)	51 (1.8)	818 (7.2)

*Abbreviations*: *F* female, *M* male, *mo* months

**Fig. 3:**
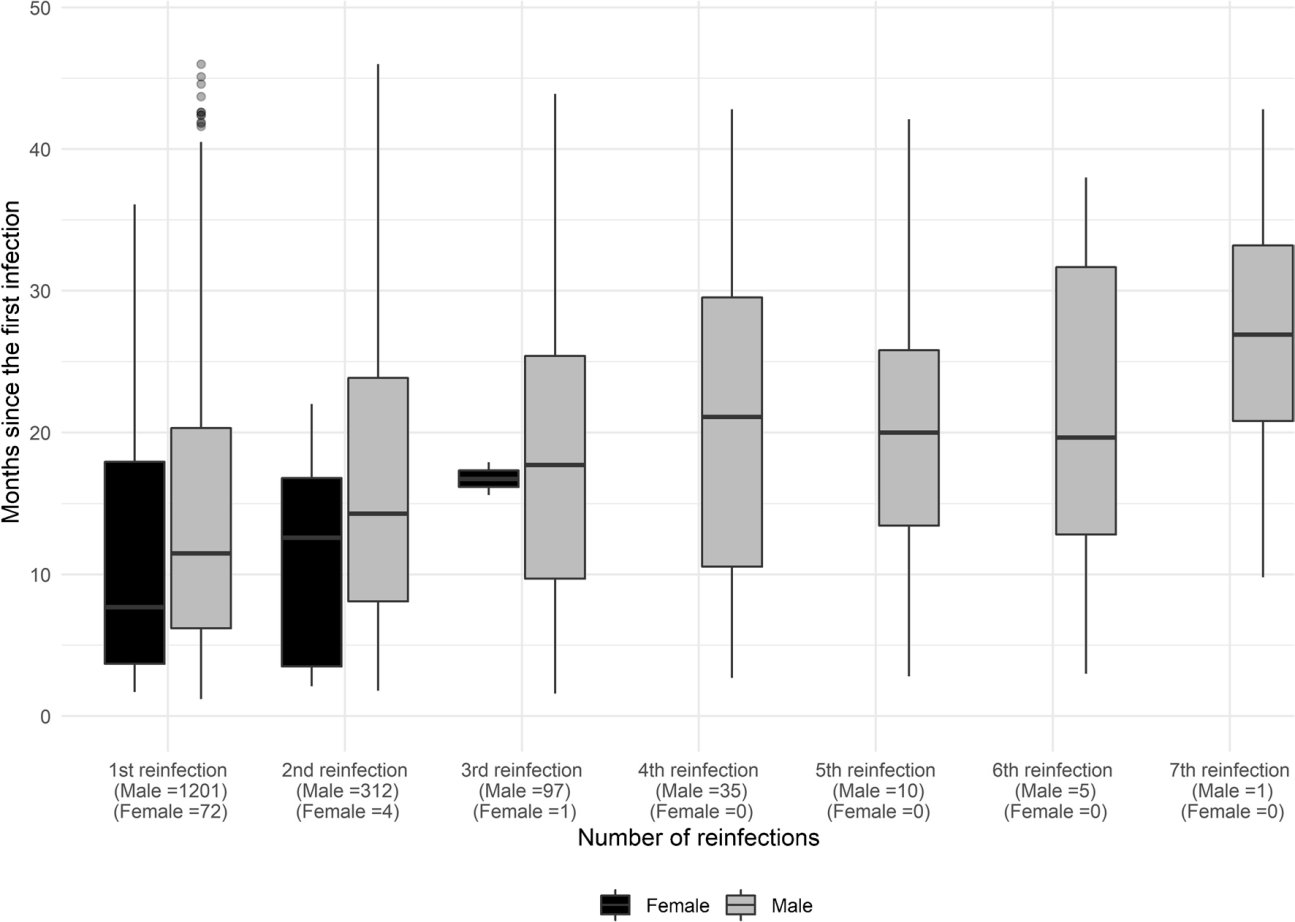
*Neisseria gonorrhoeae* reinfection episodes since primary infection by sex. Catalonia, Spain, 2016–2019

The boxplot in Fig. 3 represents the number of patients that had between one and seven reinfection episodes subsequent to the first primary infection. For each episode, the median time elapsed in months is shown. In females, the median time between one infection and the next was shorter throughout the period. The distribution of reinfections shows 8.9% (*n* = 1,273) cases with one reinfection, and 2.2% (*n* = 316) with two. In 149 cases (1.1%) there were three or more reinfections, including one patient with seven reinfections.

## Discussion

This study describes microbiological surveillance data, coinfections and reinfections of 14.251 confirmed *N. gonorrhoeae* cases in Catalonia over a four-year period. Antimicrobial susceptibility was performed on 5,377 strains. To our knowledge, this is the first study conducted in Spain to describe AMR of such a considerable amount of *N. gonorrhoeae* isolates, previously published works [8, 14] having studied far fewer strains.

The results indicate a rise in the incidence of gonococcal infection in our region in 2019 (IR = 64.7 cases/100,000 persons) compared to 2016 (IR = 30.7 cases/100,000 persons). This increase can be explained in part by improved STIs diagnosis, in addition to public health campaigns and an increase in access to rapid point-of-care testing. Furthermore, Catalonia is a reception site for sex tourism [15]. Recent studies conducted in Europe, the Netherlands and the United Kingdom [16, 17] describe that the use of substances that facilitate chemsex and other high-risk group sex activities has contributed to an increased incidence of STIs. Pre-exposure prophylaxis (PrEP) for HIV does not protect against other infections, and the use of condoms is frequently abandoned during these practices [18].

Among our study population, roughly 48.7% of cases were aged 29 or younger, and 30.5% were aged 30 to 39 at the time of diagnosis (similar to the Spanish national data profile) [19]. In our dataset, the overall ratio of male cases to female cases was 4:1, higher than the overall European ratio of 3.2:1 [6].

It is not surprising that a large proportion of our population with *N. gonorrhoeae* was coinfected with other STDs (gonorrhoea and chlamydia being the most frequent association), especially among young women [20]. This supports the findings of previous Spanish studies [21] and Spanish national guidelines that gonorrhoea infections should be treated with combination therapy (ceftriaxone or cefixime with azithromycin) to target both infections, in accordance with ECDC guidance [22]. Cervical ectopy of young women (< 25 years old) has been identified as a risk factor for chlamydia and gonorrhoea co-infection [23].

Antimicrobial surveillance data was available from the 6,292 (44.2%) culture isolates studied. Laboratory culture slightly decreased from 47.7% in 2016 to 42.9% in 2019, due to the shift from the use of bacterial culture to NAAT for gonorrhoea diagnosis. This trend has also been attested to in Canada, where 21.8% of gonococcal infections are diagnosed through culture [24]. This is a concern, as cultures are required for antimicrobial susceptibility testing and to monitor AMR. In 2015–16, the WHO’s Gonococcal Antimicrobial Surveillance Programme (WHO-GASP) documented sustained high levels of ciprofloxacin resistance [25]. And although the resistance rate to ciprofloxacin observed (57.1%) is similar to that of neighbouring European countries (53%), it is notably higher than that reported in Portugal (46%) or the Netherlands (36%) [26].

Combination therapy is now recommended for treating non-complicated gonorrhoea, given the growing resistance to cephalosporins described for *N. gonorrhoeae* in recent years [27] and treatment failures detected. Health professionals therefore need to be made aware of the importance of performing a cure test in cases where symptoms do not improve [5].

During the period of our study, we observed a decrease in the resistance rate for cefixime, especially during the later years (2018–2019). One probable explanation could be the low number of strains studied for this antibiotic, affecting the relevance of continued monitoring. It has been suggested [28] that cefixime could be reconsidered as an alternative to ceftriaxone in first-line dual therapy if this decrease is sustained over time.

The increase in resistance to azithromycin observed in Catalonia, from 6.1% in 2016 to 16% in 2019, is comparable to a similar increase in Germany (from 1.3% in 2014 to 12.2% in 2020) [29], but higher than that observed in Canada (from 0.8% in 2012 to 7.2% in 2016) [24]. Azithromycin resistance in *N. gonorrhoeae* is established and spreading in Catalonia, exceeding the 5% threshold at which WHO guidelines recommend a review to determine whether a particular antimicrobial is the appropriate treatment.

In our study, however, the rate of resistance to ceftriaxone remains low and stable, and AMR has led a number of European countries to adopt high-dose ceftriaxone monotherapy in recent years (ECDC, 2019) [30]. Therefore, close AMR surveillance following gonococcal culture is urgently needed.

Diminishing azithromycin susceptibility combined with ceftriaxone resistance is a major concern and threatens the effectiveness of the currently highly effective dual therapy regimen of ceftriaxone in conjunction with azithromycin. Even though resistance to cefixime was decreasing in our region in 2019, cefixime resistance needs to be monitored closely, particularly because gonococcal strains resistant to both cefixime and ceftriaxone continue to spread internationally [30].

We have also detected four multidrug-resistant strains that are resistant to azithromycin and ceftriaxone and cefixime, and similar evidence has emerged in other countries where the first cases of MDR-NG appeared in February 2018 (1 case in England and 2 cases in Australia) [31]. These strains are a sign of the growing threat of multidrug-resistant gonococcal strains in the context of limited therapeutic alternatives, lack of a vaccine and limited surveillance capacity in many regions of the world.

Most reinfections occurred between 3 to 6 months after the first episode and were more common in males. A recent study of 9,241 cases conducted in New Zealand showed that, of the 34% (3,151) retested at 6 weeks and 6 months, 21% tested positive [32]. Given that reinfection with chlamydia or gonorrhea is common and can lead to significant reproductive health complications, testing for reinfection after treatment might be recommended despite individual and social characteristics associated with gonorrhea patients limiting the success of this strategy [33].

The present study has a number of limitations. First, we are aware of a possible under-notification of cases, as notification is reliant on a passive system. However, the MRSC conducts a search for suspected cases reported by health professionals, which are then validated by epidemiologists and confirmed by microbiology laboratories. Second, cultures were carried out for less than half of the samples analysed, probably because of the comparative speed of diagnosis afforded by the molecular test.

The low percentage of cultures, as shown in the study, is hampering the monitoring and evaluation of AMR and facilitating the spread of infection. Lastly, antibiotic sensitivity detection rates are often low (samples arriving late to the laboratory are no longer viable, rendering antibiotic susceptibility testing impossible), indicating that a far larger number of samples is needed to build cultures and tests for antibiotic sensitivity.

## Conclusion

In Catalonia, 10% of the *N. gonorrhoeae* isolates studied between 2016 and 2019 were resistant to azithromycin. According to the WHO, resistance above 5% indicates an alert to review treatment guidelines. Resistance to ciprofloxacin, tetracycline and penicillin was relatively high and stable, while resistance to ceftriaxone and cefixime remained low; and multidrug-resistant strains of *N. gonorrhoeae* increased throughout the period. Epidemiological surveillance of *N. gonorrhoeae* must be enhanced, paying particular attention to improving the culture of strains and AMR data.

## Data Availability

All the data used in the analysis was collected during routine public health surveillance activities, as part of the legislated mandate of the Health Department of Catalonia, the competent authority for the surveillance of communicable diseases, which is officially authorized to receive, treat and temporarily store personal data on cases of infectious diseases. All data was fully anonymized.

## Abbreviations

AMR: Antimicrobial resistance
ECDC: European Centre for Disease Prevention and Control
ESC: Extended-spectrum cephalosporins
GASP: Gonococcal Antimicrobial Surveillance Programme
IR: Incidence rate
MDR: Multi-drug resistant
MRSC: Microbiological reporting system of Catalonia
NAAT: Nucleic acid amplification test
NG: *Neisseria gonorrhoeae, N. gonorrhoeae*
STI: Sexually transmitted infection
WHO: World Health Organization
XDR: Extensively-drug resistant
